# Supplementary material for: Evaluation of attraction and spatial pattern analysis of world cultural and natural heritage tourism resources in China
Source: PLoS One. 2023 Aug 8;18(8):e0289093. doi: 10.1371/journal.pone.0289093 (PMC10409290; doi:10.1371/journal.pone.0289093)
Supplement: Attachment — Submitted filename: Response to Reviewers.docx [file pone.0289093.s002.docx]

Respected reviewers and editors:

Hello! First of all, I would like to thank the reviewers and editor for their valuable comments in their busy schedule. The reviewer and editor’s suggestions have provided very useful guidance for the revision and improvement of this paper.After receiving the evaluation of the article, I was very glad and nervous. I was glad that the teachers affirmed the topic selection of the article. At the same time, I also revised the article carefully. I consulted professors and teachers for many times, and combined with the suggestions of reviewers, I carefully revised and improved the paper as follows ( the blue font refers to the suggestions of reviewers, the black font refers to the revised reply instructions, and the red font refers to the detailed modifications in the paper).

1.Methodology. The author(s) need to reconsider their research design. This paper is a technology oriented paper or a empirical paper. If it is a technology oriented paper, then they have to pay more attention to develop some new algorithm or methods such as to improve the accuracy of the prediction. On the other hand, it is an empirical paper, the author(s) need to have a conceptual framework (or some analytical models).

Thank you very much. This paper belongs to the empirical research paper, compared with the technical paper, it is more inclined to use big data in resource evaluation to prove the attraction of tourism resources. According to the scoring rules of the tourism resources evaluation part of the Chinese national standard GB/T18972-2017 "Classification, Investigation and Evaluation of tourism resources", this paper evaluates tourism resources from the aspects of resource element value, resource impact and added value. The evaluation index system is constructed to put forward a set of operable index sets. The integration of online tourist data is conducive to the quantitative evaluation or comparison of world cultural and natural heritage tourism resources, and to find out the areas that need to be upgraded and improved in terms of tourism of World heritage sites.

2.The author(s) need to think about the implication of this paper. Can the results of this paper present some new insight to the scholars or can the results give some practical suggestions to the government?

Thank you very much. By collecting the user data of China's three major OTA platforms, this paper constructs the attractiveness evaluation system of China's world cultural and natural heritage tourism resources, aiming to explore the attractiveness level of world cultural and natural heritage from different dimensions, and explore the attractiveness characteristics of tourism resources from space. There are two main innovations:

1. Data innovation. The data in this paper come from the real ratings and comments of tourists in different aspects of the three well-known tourism platforms in China, which ensures the authenticity, reliability and availability of the data. The data source is not limited by region, and the real views of tourists can be understood more comprehensively.

2. Method innovation. According to the national standard GB/T18972-2017 "Classification, Investigation and Evaluation of Tourism Resources" issued by China, this paper divides China's world cultural and natural heritage tourism resources into five main categories according to the specific causes and status of tourism resources: Geographical landscape, aquatic landscape, biological landscape, architecture and facilities, and historical sites are subdivided into ten sub-categories. Using the kernel density and the Moran index from a finer dimension, the different attractiveness of different resource types is compared in geographical space.

The innovation of the above research content can provide a new research perspective for scholars. This study reflects the attractiveness of tourism resources of China's world cultural and natural heritage in different dimensions. In practice, it can also provide guidance and suggestions for scenic spots with different resource types to strengthen tourism attraction construction according to their disadvantage dimensions.

3.For the kernel density analysis, because I did not obtain the copyright of the map, I deleted the previous drawing, please understand, thank you.
